# Development, feasibility and usability of an app for the correct nutrition of children and adolescents with acute lymphoblastic leukemia

**DOI:** 10.3389/fonc.2025.1577933

**Published:** 2025-10-02

**Authors:** Andrea Zibaldo, Pio Stellato, Gaia Sepe, Diana Fenicia, Armando Scognamiglio, Francesco Zibaldo, Rosanna Parasole, Giuseppe Menna

**Affiliations:** ^1^ Department of Oncology, Hematology and Cellular Therapy, Santobono-Pausilipon Children’s Hospital, Naples, Italy; ^2^ Pediatric Dietetics and Clinical Nutrition Unit, Department of Clinical Management Staff, Santobono-Pausilipon Children’s Hospital, Naples, Italy; ^3^ School of Science, Engineering and Health, University of Naples “Parthenope”, Naples, Italy

**Keywords:** acute lymphoblastic leukemia, app development, cancer, family compliance, nutrition, pediatric, survey

## Abstract

**Introduction:**

Proper nutrition during cancer treatment is crucial for children and adolescents with acute lymphoblastic leukemia (ALL), as malnutrition can impact treatment tolerance, survival, and quality of life. In ALL specifically, malnutrition at diagnosis has been associated with increased risk of infections, treatment delays, and poorer survival, while maintaining adequate nutritional status can improve treatment tolerance and long-term outcomes.

**Materials and methods:**

We developed and tested the “Hematognam” app, which provides caregivers with dietary guidance for children and adolescents undergoing chemotherapy. This study evaluates the app’s feasibility and usability among families of children and adolescents with ALL over a three-month period.

**Results:**

The app received high satisfaction ratings, which suggests its user-centric design and relevance in managing complex dietary challenges associated with treatment.

**Discussion:**

These results align with previous studies demonstrating the potential of digital health tools to enhance treatment compliance and overall well-being in vulnerable populations. By addressing key nutritional challenges faced by children and adolescents with ALL - such as appetite loss, food aversions, gastrointestinal side effects, and food safety concerns - the "Hematognam" app offers a complementary tool to existing dietary counseling, supporting both families and clinicians in daily nutritional management. The development and implementation of the “Hematognam” app proved feasible and effective in addressing the nutritional needs of children and adolescents with ALL during chemotherapy.

## 1 Introduction

The long-term complications associated with multimodal therapy for malignancies - which include chemotherapy, surgery, and radiation - expose a significant proportion of cancer survivors at serious risk for life-threatening chronic medical conditions. Many of these debilitating conditions, such as cardiovascular disease, type 2 diabetes, metabolic syndrome, and even secondary malignancies, may be exacerbated by nutrition - related problems that first manifest during therapy (1, 2).

Proper nutritional status during cancer therapy is crucial for various outcomes, including overall survival, treatment tolerance, susceptibility to infection, and quality of life (3, 4). In children with acute lymphoblastic leukemia (ALL), nutrition plays an even more critical role. Malnutrition at diagnosis has been associated with increased treatment-related complications, such as higher risk of neutropenia and infections, treatment delays, and poorer survival outcomes (4–6). Conversely, maintaining adequate nutritional support can improve tolerance to chemotherapy, support growth and development, and contribute to better overall prognosis. These considerations underline the importance of integrating structured nutritional guidance into supportive care for pediatric patients with ALL. Due to the complex interaction between cancer, physiological requirements for growth and development, the effects of multimodal therapy, and treatment-related toxicities, children and adolescents with cancer are at high risk of developing nutritional deficiencies (7, 8). However, significant gaps persist in the scientific community’s and healthcare providers’ approach to studying malnutrition in this population (6, 9). Additionally, the intensive effort required for cancer treatment, worsening by the lack of a universal consensus to identify children and adolescents at risk of malnutrition, often leads to this critical issue being underestimated and unaddressed in many hemato-oncology pediatric centers (2, 3, 10).

The incidence of malnutrition during cancer treatment varies greatly between disease types and modes of intervention, with undernutrition reported in 0% to 70% of cases and overnutrition in 25% to 75% (11, 12). The presence of evident malnutrition at diagnosis has been shown to be a negative prognostic factor, associated with high rates of neutropenia and febrile neutropenia (5).

Diet-related problems can affect the quality of life (QoL) of survivors and predispose them to other chronic diseases (2), underscoring the need for scientific management and nutritional support for this population. Literature indicates that parents need comprehensive and regular information on nutrition, while pediatric oncology nurses play a significant role in assessing, training, and monitoring these children and adolescents (13). Experiences from some institutions have demonstrated the usefulness of digital support for these families in increasing compliance and nutrition education (14).

At the Department of Oncology, Hematology and Cellular Therapy of the Santobono-Pausilipon children’s hospital in Naples, Italy, the nursing staff is actively involved in providing nutritional education to support families thrust into a sudden and uncertain reality. These families must manage medication regimens, learn new medical terms and protocols, control strong emotions, and adhere to a proper diet, which may be unfamiliar to them. Hence, the need to develop a tool to support children, adolescents and caregivers, simplifying their approach to diet, reducing doubts, encouraging autonomous management (self-care), reducing infection risk, and ensuring constant support from the nursing-medical-dietetic team. Children and adolescents with ALL face multiple nutritional challenges during therapy, including reduced appetite, taste alterations, food aversions, nausea, gastrointestinal toxicities, and metabolic complications such as undernutrition or overweight. In addition, families must cope with food safety restrictions according to drugs interactions and foodborne illness risk. Addressing these challenges requires clear, accessible, and phase-specific guidance, which was a central aim of the "Hematognam" app.

### 1.1 Objectives

The primary objective of this project is to develop an app to guide the correct nutrition of children and adolescents with cancer.

Secondary objectives include:

- Evaluating the feasibility and usability of the app;
- Improving compliance of children, adolescents and families with a proper diet during chemotherapy and drug induced neutropenia;
- Offering clear information about drugs compatibility with diet and lifestyles.

## 2 Materials and methods

This observational, single-center study was conducted at the Day hospital and Hemato-Oncology Units of the Santobono-Pausilipon Children’s Hospital in Naples. A total of 20 families of children and adolescents undergoing chemotherapy for Acute Lymphoblastic Leukemia (ALL) were recruited since these were the most common and treated in the center at the time of the study. Participants used the “Hematognam” app for a three-month period, during which data on app usage and feedback on usability were collected.

### 2.1 Study design

The study was designed as observational, non-pharmacological, monocentric. The app was developed following a thorough literature review focused on the nutritional needs of children and adolescents undergoing chemotherapy. The app provided caregivers with nutritional recommendations tailored to the different treatment stages (induction, consolidation, reinduction, and maintenance). After minimum three months and maximum four months of app use, families were asked to evaluate the usability and effectiveness of the app through an anonymous questionnaire distributed and collected by a member of the research team. No formal sample size calculation was performed, as the study was designed as an exploratory feasibility and usability assessment. A total of 20 families (20 pediatric patients and 20 parents/caregivers) was included, corresponding to the consecutive eligible cases during the recruitment period. Prior usability research suggest that 15–20 participants per user group are sufficient to detect the majority of major usability problems (15, 16). Furthermore, with 20 participants per group, the precision of quantitative usability measures is acceptable: assuming a standard deviation of approximately 12.5 on the System Usability Scale (SUS), a sample of 20 yields a 95% confidence interval with a half-width of about 5.5 points, which is adequate for a pilot study (17).

### 2.2 Participants

The study included 20 families of children and adolescents diagnosed with ALL undergoing chemotherapy at the Department of Oncology, Hematology and Cellular Therapy of the Santobono-Pausilipon Children’s Hospital. Families with children or adolescents aged 1–17 years diagnosed with ALL, undergoing chemotherapy and owning a digital device (smartphone or tablet) with internet access were included in the study. The app was designed for bilateral use, allowing either parents/legal guardians or children/adolescents (depending on age and family preference) to access nutritional information and recommendations. In families with younger children, parents were the primary users, while older children and adolescents could also directly access the app.

Families who did not consent to participate or lacked access to a digital device were excluded from the study​.

### 2.3 App development

The development of the app involved collaboration between a multidisciplinary team, including two pediatric nurses (A.Z. and D.F.), three medical doctors (G.M., R.P. and P.S.), one study coordinator (G.S.), one dietitian (A.S.), and an app developer (F.Z.). The app was designed with a user-friendly interface that allowed caregivers to:

- Enter basic information about the child/adolescent (e.g., age, gender, treatment stage);
- Access tailored nutritional advice based on the child/adolescent’s current phase of chemotherapy;
- Submit questions to the medical team via a Frequently Asked Questions (FAQ) section​.

Although usability feedback was collected from parents/legal guardians, the app interface was designed to be accessible also to adolescents, supporting shared use between patients and their caregivers.

The app featured three main screens: a) A welcome screen where caregivers would input socio-demographic data (gender and age) about their child and themselves; b) A menu for selecting the chemotherapy phase; c) A screen displaying allowed and discouraged foods and the rationale behind these; basic hygiene rules for food preparing; d) A screen displaying the drugs received by the child/adolescent during the specific treatment phase and the description of the incompatibility with certain foods and the rationale behind it. For younger children (<6 years), the app was used exclusively by parents/legal guardians, while older children and adolescents could access it directly, depending on family preference.

### 2.4 Data collection

A member of the research team approached the parent of a child/adolescent looked after at the Day hospital and Hemato-Oncology Units of the Department of Oncology, Hematology and Cellular Therapy of the Santobono-Pausilipon Children’s Hospital from 05/07/2023 to 17/11/2023. After explaining the purpose and modalities of participation, if parents agreed to be involved in the study, the written informed consent form was obtained. For study purposes, feedback questionnaires were completed by parents/legal guardians, but children and adolescents could also use the app in daily practice. The research team supported the child/adolescent and the caregivers in downloading the App and in the registration steps. The app tracked: number of accesses; FAQs submitted; requests made by caregivers, linked with demographic data (caregiver’s and child/adolescent’s age and gender, treatment stage). Participants were eventually asked to rate the app’s usability on a scale of 1 to 10 after three/four months of use.

Feasibility was assessed through monitoring number of app accesses, response times to family queries and tracking any technical issues reported during the study​.

Usability and feasibility were assessed using an adapted questionnaire that has been previously applied in studies evaluating digital health tools in pediatric oncology (18). The questionnaire covered domains such as ease of use, clarity of information, satisfaction, and recommendation. While not a formally validated international instrument, it was structured to capture key usability indicators relevant to this feasibility study.

### 2.5 Statistical analysis

Data were collected through the app, which recorded the number of accesses, questions submitted, and requests made by the users. Statistical analysis was performed using descriptive statistics for demographic data, including median, interquartile range, and proportions for categorical variables. Missing data were addressed by contacting families, and any unrecoverable data were treated as missing.

### 2.6 Ethical considerations

​The study was conducted in compliance with ethical guidelines, including the Declaration of Helsinki, the Good Clinical Practice (GCP) guidelines and the European General Data Protection Regulation (GDPR). Families provided informed consent to be involved in the study and the privacy, and data were anonymized using unique access codes. The app’s database was password-protected and accessible only to the principal investigators​. The study protocol, the data collection instrument, and the consent forms were approved by the Ethics Committee of the IRCCS Pascale-AORN Santobono-Pausilipon (code 15/22 OSS SP).

## 3 Results

### 3.1 Demographics and usage patterns

The average age of the caregivers was 40.7 ± 5.8 years (range 32-52), while the average age of the children/adolescents was 9.7 ± 4.7 years (range 1-17)​. A total of 65% of the children/adolescents had been diagnosed with ALL-T, while 35% had been diagnosed with ALL-B. Notably, parents of children and adolescents with ALL-T accessed the app more frequently (mean accesses 23.7) compared to those with ALL-B (mean accesses 8.1). Twelve of the 20 families made minimal use of the app, with a mean of 2.4 ± 4.9 accesses (range 0-19)​. Caregivers made an average of 13.5 ± 15.5 (range 0-57) accesses to the app. Only one caregiver did not access the app. The number of accesses was more frequent in parents of children/adolescents undergoing the “Consolidation” phase (average 5.7 ± 8.6 accesses; range 0-30) (**Table 1** reports the total number of accesses recorded by caregivers and children/adolescents in each chemotherapy phase, whereas in the text we describe the average number of accesses per family (mean ± SD, range).

**Table 1:** Total number of accesses recorded for caregivers and children/adolescents across the different phases of chemotherapy.

Phase of chemotherapy	Number of caregivers’ access	Number of child/adolescent’s access
Induction	21	71
Consolidation	20	113
Re-induction	6	55
Maintenance	1	32
Total of access	48	271

### 3.2 Feasibility and usability feedback

Parents rated the app’s overall quality high, with an average score of 9.1 ± 1.1. Most parents (65%) indicated that the app significantly improved their son/daughter’s health and well-being (mean score of 9.45 ± 0.8). Usability was also rated positively, with parents scoring the ease of use at 9.5 ± 0.9 and the quality of information at 9.2 ± 1.1. Results of the survey are summarized in **Table 2**.

**Table 2:** Survey on feasibility and usability of the app "Hematognam".

Questions	Score*					
	6	7	8	9	10	Average, SD
	N (%)	N (%)	N (%)	N (%)	N (%)	
Is the Hematognam app of good quality?	-	1 (5)	7 (35)	2 (10)	10 (50)	9.1 ± 1.1
Was the Hematognam app useful for your son/daughter’s health and well-being?	-	–	4 (20)	3 (15)	13 (65)	9.45 ± 0.8
Has the Hematognam app met your nutritional information needs?	1 (5)	–	6 (30)	4 (20)	9 (45)	9 ± 1.1
If another parent needed a similar app, would you recommend it?	–	–	3 (15)	2 (10)	15 (75)	9.6 ± 0.75
Did the information provided by the Hematognam app help you manage your health-related nutrition problems more effectively?	–	2 (10)	6 (30)	1 (5)	11 (55)	9.1 ± 1.1
Did the use of the Hematognam app help your son/daughter to comply with the guidelines on proper nutrition during illness?	-	1 (5)	7 (35)	3 (15)	9 (45)	9 ± 1.1
If you were looking for a food app, would you use the Hematognam app again?	-	–	3 (15)	5 (25)	12 (60)	9.45 ± 0.76
Ease of access	2 (10)	2 (10)	3 (15)	2 (10)	11 (55)	8.9 ± 1.5
Ease of use	–	2 (10)	–	3 (15)	15 (76)	9.5 ± 0.9
Graphical interface (colors, screens, links, etc.)	-	1 (5)	3 (15)	6 (30)	10 (50)	9.2 ± 0.9
Privacy and security	–	–	1 (5)	2 (10)	17 (85)	9.8 ± 0.5
App free	–	–	1 (5)	1 (5)	18 (90)	9.8 ± 0.5
Download size and time	-	1 (5)	3 (15)	1 (5)	15 (75)	9.5 ± 1
Clarity of information provided	-	1 (5)	4 (20)	5 (25)	10 (50)	9.2 ± 1
Organizing the app and finding the information you need	–	3 (15)	2 (10)	4 (20)	11 (55)	9.1 ± 1.1
Quality of information included		1 (5)	6 (30)	6 (30)	7 (35)	8.9 ± 1
Categories of food included	–	1 (5)	5 (25)	4 (20)	10 (50)	9.1 ± 1
Scientific evidence of the included information	–	–	5 ( 25)	5 (25)	10 (50)	9.2 ± 0.8
Technical information provided by staff	–	1 (5)	4 (20)	5 (25)	10 (50)	9.2 ± 0.9
Achievement of initial expectations	–	–	5 (25)	6 (30)	9 (45)	9.2 ± 0.8
Overall satisfaction level of the app	–	–	4 (20)	5 (25)	11 (55)	9.3 ± 0.8

*No score under 6 was ever assessed.

### 3.3 Challenges and area of improvement

Despite the app’s high usability scores, several areas for improvement were identified. For instance, caregivers reported difficulties with accessing the app due to forgotten credentials and requested a more streamlined login process such as facial recognition or fingerprint. There was also a demand for additional features, such as the ability to consult a nutrition expert directly through the app​, adding weekly meal plans, supporting all operating systems​. In **Table 3** are summarized the caregivers’ comments and advice for improvement.

**Table 3:** Caregivers’ comment and advice for app improvement.

• Provide examples of weekly menus. • Add an open forum with a nutrition specialist. • Integrate the app with dietary advice in case of high triglycerides/transaminases or hyper/hypoglycemia. • Facilitated access in case of forgetting the login credentials. • Make available for all operating systems, facilitate access by remembering credentials perhaps with facial recognition, include in app window to submit questions. • I think it is healthier to convey the concept of taking care of oneself and letting others help you. • Have the opportunity to ask questions to the expert nutritionist on the missing topics. • Search by keywords would be useful. • Expand the section on the way of taking medications and any side effects. App that I would definitely recommend.

## 4 Discussion

The “Hematognam” app was well-received by caregivers, who found it to be a valuable tool for managing the nutritional needs of their children/adolescents undergoing chemotherapy. The high usability scores reflect the app’s user-friendly design and relevance to the families’ needs. The app’s tailored nutritional advice helped to reduce the uncertainty associated with managing a child/adolescent’s diet during cancer treatment, improving dietary compliance and reducing stress for caregivers. At our center, clinical nutrition support is provided through dietary counseling by pediatric dietitians and oncology nurses. However, these interventions are often fragmented and may be difficult for families to implement consistently in daily life. The "Hematognam" app was therefore designed to complement routine nutritional counseling by offering families a standardized, easily accessible, and continuously available tool both at home and during hospitalization. In this way, the app bridges the gap between periodic in-person consultations and the ongoing daily dietary decisions families must make.

It has already been described in the literature that the use of apps improves patient adherence and compliance (19, 20). The app allows users to have useful information and reveal themselves as official sources of information. In fact, patients often use unofficial sources of information, such as websites or unverified apps, which puts their health at risk and does not comply with the treatment plan (21).

The presented app, through the guarantee of an operator who responds quickly to user’s doubts, is a valid alternative to telemedicine. Telemedicine, especially after the COVID-19 pandemic, has proven to be an excellent public health strategy as it allows a quick, easy and inexpensive way to contact healthcare professionals (22, 23).

However, the study identified areas that could enhance the app’s functionality and user experience. Implementing suggestions such as enabling other operating systems compatibility, improving login procedures, and expanding the FAQ section would likely increase engagement and satisfaction. Moreover, the integration of direct access to a nutritionist or medical expert could further support families in managing complex dietary issues during treatment, reducing caregivers’ anxiety and fear of making dietary mistakes. Feedback from families also provided insight into which features they considered essential in a nutrition app. Caregivers particularly valued the clarity of dietary information, food safety guidance, and drug–food interaction alerts. They suggested the addition of weekly meal plans, direct access to a nutritionist, simplified login (e.g., biometric authentication), and compatibility across different operating systems. These elements represent priorities for future app iterations and underline the importance of co-design with end-users.

### 4.1 Limitations

The present study has some potential methodological limitations that should be taken into consideration when interpreting the results. First, the recruitment of the sample was conducted in only one hospital and the sample size is small; therefore, the aim of the study was to test usability and feasibility of the app and for this purpose the sample recruited appears sufficient. However, a wider usage of the app to a broader population could confirm our observations, since it is possible that the findings may not be generalized to the whole population. Second, information regarding usability and feasibility was gathered through a questionnaire collected by a member of the research team and could not be verified based on direct observation, leading to a potential recall bias. Third, caregivers may have answered questions in a socially desirable way. However, these risks are reduced guaranteeing that data are collected anonymously. In addition to these described limitations, we are confident that the results give relevant and valuable information on this innovative app.

Another limitation is that usability and feasibility were assessed through an adapted questionnaire rather than a formally validated international tool, although the instrument had been previously used in similar feasibility assessments.

## 5 Conclusion

The development and implementation of the “Hematognam” app proved feasible and effective in addressing the nutritional needs of children and adolescents with ALL during chemotherapy. The app received high satisfaction ratings, which validates its user-centric design and relevance in managing complex dietary challenges associated with treatment. These results align with previous studies demonstrating the potential of digital health tools to enhance treatment compliance and overall well-being in vulnerable populations. Given the increasing role of digital health tools in pediatric oncology care, our research provides valuable insights also for nurses, clinicians, researchers and healthcare stakeholders aiming to enhance supportive care for children with cancer.

### 5.1 Critical suggestions for improvement

Expand accessibility: To ensure broader applicability, the app should support additional operating systems and streamline login processes through biometric authentication (e.g., facial recognition or fingerprint scanning).

Enhance features: Incorporating weekly meal plans and an interactive FAQ section with access to nutrition experts would further elevate user experience and utility.

Strengthen educational components: Expanding sections on medication compatibility and food-related risks can empower caregivers to make informed decisions.

By implementing these enhancements, the “Hematognam” app could become an indispensable tool for nutritional management, improving outcomes and quality of life for children with ALL.

### 5.2 Future perspectives

Extend the app’s usability to all the families and children/adolescents with cancer, introducing more drugs and adjusting the various treatments according to the specific needs.

## Data Availability

The original contributions presented in the study are included in the article/supplementary material. Further inquiries can be directed to the corresponding author.
